# Fungal colonisation on wood surfaces weathered at diverse climatic conditions

**DOI:** 10.1016/j.heliyon.2023.e17355

**Published:** 2023-06-20

**Authors:** Faksawat Poohphajai, Olena Myronycheva, Olov Karlsson, Tiina Belt, Lauri Rautkari, Jakub Sandak, Ana Gubenšek, Polona Zalar, Nina Gunde-Cimerman, Anna Sandak

**Affiliations:** a Department of Bioproducts and Biosystems, School of Chemical Engineering, Aalto University, P.O. Box 16300, 00076, Aalto, Finland; b InnoRenew CoE, Livade 6a, 6310, Izola, Slovenia; c Luleå University of Technology, Wood Science and Engineering, Forskargatan 1, 931 87, Skellefteå, Sweden; d Andrej Marušič Institute, University of Primorska, Titov trg 4, 6000, Koper, Slovenia; e Production Systems, Natural Resources Institute Finland (Luke), Tietotie 2, 02150, Espoo, Finland; f University of Ljubljana, Biotechnical Faculty, Department of Biology, Večna pot 111, SI-1000, Ljubljana, Slovenia; g Faculty of Mathematics, Natural Sciences and Information Technologies, University of Primorska, Glagoljaška 8, 6000, Koper, Slovenia

**Keywords:** Fungal infestation, Colonisation rate, Natural weathering, Ascomycetes fungi, Mould, Biofilm formation

## Abstract

Natural weathering test at two different European climatic zones were conducted to investigate simultaneously both, the fungal colonisation and weathering process of Scots pine wood (*Pinus sylvestris* L.). The hypothesis was that the wood performing differently in various climate conditions might affect fungal infestation. The colour changes, wettability, and glossiness were measured as indicators of weathering progress of wood together with an assessment of fungal diversity. Different intensities in weathering, occupancy, and colonisation of fungi on wooden surface were detected. A higher number of fungal species was found on wood exposed to the warm temperate climates compared to subarctic or boreal climates. The dominant fungal species in both locations were from the genera *Cladosporium* and *Aureobasidium.*

## 1 Introduction

Wood is a sustainable bio-based material that plays a significant role in fulfilling the United Nations' sustainable development goals, particularly, SDG 12 (Responsible Consumption and Production), 13 (Climate Action), and 15 (Life on Land) [1]. The use of wood products in construction can significantly reduce embodied carbon emissions through substitution and storage. Reusing or recycling wood as well as proper management of waste produced during construction and demolition can decrease the volume of virgin wood that needs to be harvested from forests. In combination with clean manufacturing technologies and innovative designs, wood can deliver low carbon and sustainable products for the building sector. However, despite the bright and renewable perspectives, wood readily decomposes by natural biotic and abiotic processes [2]. These can be slowed down if the material is adequately protected and maintained in buildings and constructions [3]. Deterioration processes occur when wood that is exposed to the outdoors, undergoes degradation due to weathering processes caused by the mutual action of environmental (abiotic) factors and biological attacks [4]. This process results in surface discolouration, loose fibres, raised grain, checks, cracks, and roughening/erosion [4,5]. The surface of weathered wood is often colonised by fungi and lichen [4]. The infestation by fungi can lead to further discolouration of weathered wood and affect the quality of the living environment [5].

Microbial colonisation is one of the most important aspects of the degradation process, which leads to a change in the aesthetic appearance and furthers material deterioration [6]. It causes economic loss due to an increase in maintenance costs and significantly limits the service life of wood [[6], [7], [8]]. Fungal degradation of wood is complex; it can be influenced by the properties of wood (endogenous factors) and abiotic/biotic environmental influences (exogenous factors) [3,[9], [10], [11], [12], [13]]. The composition and concentration of airborne fungal spores in the atmosphere are influenced by different factors such as geographic location, meteorological factors, seasonal climatic factors, air pollutants, vegetation, and human activities [[14], [15], [16], [17]]. Correlation between the site-specific climate and the hazard for decay of wood was proposed by Scheffer [18] and is commonly recognised as the “Scheffer index” (SCI). In his study, Scheffer developed a single formula for determining the potential decay intensity at any geographic location within the United States territory. The initial work focused on wood exposed to exterior conditions above the ground. Scheffer used temperature and distribution of rainfall as variables in the model. The study showed that the value of the climate index is directly correlated with the decay hazard of wood [18]. Corresponding climate index maps were prepared for other application fields and/or locations following Scheffer's concept, e.g. a hazard map for the contiguous United States presented in the Wood Handbook by Forest Products Laboratory [19], in Canada [20], decay hazard classification for exterior aboveground wood in China [21], exterior aboveground wood in Korea [22], and in Switzerland [12]. The potential risk of wood decay in Norway was studied using the present and predicted climate data. An increase in air temperature and SCI for the period 2021–2050, compared with the period 1961–1990 was detected [23]. Brischke and Rapp, 2008, conducted a 7-year double layer field trial at 23 different European test sites under various exposure conditions. Their findings revealed a weak correlation between the decay rate and cumulative Scheffer index values. To address this issue, they introduced a “Dose-response model” that enables the measurement of the impact of decay on the toughness and lifespan of various wood materials [24]. The climate change that has happened over the past 55 years and increasing rot risk for wooden buildings in the cities of Oslo and Bergen in Norway have been revealed [25]. In Europe, a research group at VTT developed climate data that considers exposure conditions across the continent as part of the Woodexter project. This approach involves evaluating the level of climate exposure based on various factors, including geographical location, local exposure conditions, sheltering, ground distance, and detailed solutions. They use a dose-response model to assess the climate conditions in selected places in Europe, taking into account the ambient microclimatic conditions, particularly moisture conditions, which are critical for wood durability. The resulting classification of climate conditions in Europe is based on different climatic exposure areas or zones, including Northern European (north and south), Continental, Atlantic (north, middle, and south), and Mediterranean zone (wet and dry), providing relative values for decay risk in different parts of Europe [26].

Weathering and fungal degradation of wood have been intensively studied for several decades. However, the results obtained from different climate conditions at different locations for varying exposure periods are hardly comparable and they cannot be directly transferred to other locations. Moreover, the individual factors involved in the weathering process of wood rarely act separately, but rather operate together and frequently with a high synergy [4]. Thus, the correlation between climatic conditions and weathering process of wood, including intensity and dynamics of fungal colonisation on weathered wood is still insufficiently understood. Natural weathering tests performed at various locations with different climate conditions allows for an understanding of the correlation between dose and response [[27], [28], [29]]. In this context the dose corresponds to the specific weather conditions in different climatic zones. Correspondingly, the response of the materials includes both, degradation of the wood surface as well as fungal colonisation. The growth of mould on the wood surface, even if not directly resulting in decay is considered as another critical factor that determines the aesthetic appeal as well as health hazard for building users. A dedicated model linking climate conditions with mould growth has been proposed by VTT [30]. The optimal conditions for the growth of microorganisms on the surface were identified as related to the air temperature as well as to relative humidity and related liquid water condensation. It varies for different building materials as well as infesting microorganisms.

The site-specific climate highly influences microbial colonisation, fungal growth, and decay on wood. Consequently, diverse protection techniques are needed for wood exposed to varying climate conditions [18]. Numerous surface treatment solutions have been developed to protect wood against weathering-induced deterioration. The application of a coating is one of the most common methods to prevent wood alteration. Although the application of coatings has been widely used for wood protection for a long time, the degradation of the coating layer due to weathering is often highly problematic. Moreover, an important drawback of modern coating formulations is their chemical composition. Many surface finishing products contain toxic substances that might have a negative impact on the environment and/or can be harmful to living organisms, including humans [31]. An alternative coating system is currently under development within the frame of the ARCHI-SKIN project. It is driven by the bioinspired approach of capturing and exploiting properties that have evolved in Nature. The living coating system is based on controlled and optimised fungal biofilm which is meant to be used for surface protection [32].

This study is a part of the above scientific initiative (ARCHI-SKIN project) focused on developing novel bioinspired solutions for material protection. The new coating system is based on the controlled and laboratory optimised fungal biofilm formation. What is not known is the effect of the changing substrate properties due to natural weathering on the bioreceptivity or microbial colonisation rate. The exposure of biofilm to alien species along the service life also has not yet been properly researched. The goal of this study was to understand the relationship between specific weather conditions (causing natural weathering) and colonisation of fungal species infesting the surface of the virgin wood. An assessment of the portfolio of fungal species that can thrive on exposed surfaces and understanding their interactions with material is crucial to identify optimal strains with the highest protective potential.

## 2 Materials and methods

### 2.1 Experimental samples and natural weathering test

**Fig. 1:**
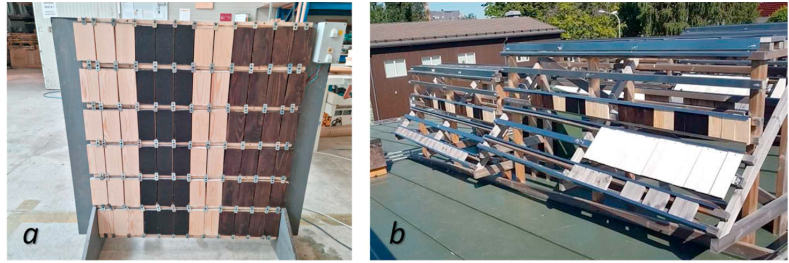
Exposure stands at weathering test site in Izola, Slovenia (a) and in Skellefteå, Sweden (b).

Forty-two blocks of wood made of kiln dried Scots pine (*Pinus sylvestris* L.) sapwood (Stenvalls Trä AB, Piteå, Sweden) with dimensions of 150 mm × 75 mm × 20 mm (length × width × thickness, respectively) were prepared as experimental samples from a single batch of timber. The exposure stand was constructed based on the standard ISO 2810:2020 [33]. Fig. 1 shows the sample sets installed on the stand before exposure. Scots pine was selected, since it is recommended as the reference test substrate according to standard procedure for natural weathering [34]. Samples were not sterilized before the experiment; however, the surface of the wood was swabbed before the test. During this swabbing, only spores of *Penicillium* spp, which is a common indoor coloniser were detected. However, after exposure, the presence of any fungi belonging to this taxon was not observed. These results suggest that the exposure conditions did not promote the growth and colonisation of *Penicillium* spp on the wood material. The samples were conditioned under controlled climate conditions at 20 °C and 65% relative humidity in the climatic chamber until constant mass was reached. Two distinct climate zones in Europe were selected as exposure sites: Izola (Slovenia, 45°32'12.98″N, 13°39'42.98″E) representing warm temperate climates (Cfa) with hot, fully humid summers and cool to mild winters; and Skellefteå (Sweden, 64°45'2.41″N, 20°57'10.04″E) representing subarctic or boreal climates (Dfc) with very long, cold freezing winters, and short, cool summers [35,36]. Locations were selected to represent diverse scenarios of deterioration mechanisms and corresponding changes to the aesthetical performance of wood exposed to natural weathering. The wood samples were exposed simultaneously at both study locations to natural weathering for 12 weeks, starting from July 1st until September 30th, 2021. This period was selected since changes in wood structure, caused by natural weathering are more intensive during the summer months in comparison with autumn and winter [37]. Moreover, the weather condition during this period promotes microbial growth. Eighteen wood samples for each location were securely fastened to the exposure stand using stainless steel attachments, with their radial surfaces positioned vertically to simulate a typical building façade configuration. The exposure stand was constructed in accordance with ISO 2810:2020 standards and positioned to face the southern direction, which is considered as the most severe exposure site [38]. Three reference wood samples were stored in the dark under controlled climate conditions at 20 °C and 65% relative humidity in the climatic chamber. Three replicate samples were collected from the stand every second week to determine the progress of deterioration. These samples were conditioned in the climatic chamber (20 °C, 65% RH) for further characterisations. Consequently, samples exposed for 0, 2, 4, 6, 8, and 12 weeks were gathered at the end the experimental campaign at both study locations. It is important to note that once the natural weathering process had concluded, all the wood samples obtained from Skellefteå, Sweden were carefully packed into vacuum-sealed bags before being transported to the InnoRenew CoE laboratory in Izola, Slovenia for further analysis. The use of vacuum-sealed bags to create a modified atmosphere by removing oxygen was employed for the purpose of transportation. It is highly effective in inhibiting biological activities and preventing fungal growth and is commonly used to extend the shelf life of food [[39], [40], [41]]. The vacuum-sealed bag also helps to keep wood samples from coming into contact with the air and prevents moisture exchange between the wood and the surrounding atmosphere.

### 2.2 Weather data

Hourly local weather conditions were recorded during the exposure period and were used to interpret the fungal growth kinetics. The weather data during the natural weathering test in Skellefteå were obtained from the Skelleftea Meteorological Station (ID: SWE00140394), located 1 km from the study site, and from the Portorož-Letalisce Meteorological Station (ID: SIE00115166), located 6.3 km from the study site in Izola (https://www.meteoblue.com). The study collected five hourly weather data parameters, including average temperature, precipitation, wind direction, wind speed, and relative humidity (RH). These weather data parameters were essential for understanding the environmental conditions that affected fungal growth during the natural weathering tests.

The Scheffer Climate Index (SCI) was used to characterise the site-specific relative hazard for above-ground wood decay of the exposure locations over a period of 30 years (1992–2021). To achieve this, we utilised the Scheffer Climate Index (*SCI*), which was determined by Equation (1) according to Scheffer, 1971 [18] as follows:

(1) $$\text{SCI}=\sum_{m=\text{Jan}}^{\text{Dec}}\left[\left(T_{m}-2\right)\left(D_{m}-3\right)\right]/16.7$$

where: *T*_*m*_ denotes the monthly average temperature (°C) and *D*_*m*_ is the number of days in a month with more than 0.25 mm of rainfall.

The SCI values were classified into three index levels based on their magnitude [18]. Values less than 35 indicated the least favourable conditions for decay, whereas values between 30 and 65 indicated intermediately favourable conditions. Values greater than 65 were indicative of the conditions most conducive to decay.

### 2.3 Analysis of the fungi present on wood surfaces

#### 2.3.1 Cultivation

Sampling of the wood surfaces was performed in accordance with the standard SS-ISO 16000–21:2015 [42]. The procedure involved using a dry sterile cotton swab to mechanically swab an area of approximately 25 mm × 25 mm on the upper surface of each outdoor exposed wood sample. The swab was then streaked out on malt extract agar (MEA) that was supplemented with 100 μg/ml chloramphenicol (Sigma Aldrich). The plates were then incubated in a growth chamber at 25 °C for a period of 7 days. Pure cultures were obtained from all colonies with different morphologies that grew on MEA after the seven-day incubation period. Selected representative isolates were identified and deposited in the Ex Culture Collection of the Infrastructural Centre Mycosmo, MRIC UL, Slovenia (http://www.ex-genebank.com) at the Department of Biology, Biotechnical Faculty, University of Ljubljana.

#### 2.3.2 Molecular identification

Genomic DNA of the isolates was extracted after mechanical lysis in CTAB buffer according to the protocol described by Gerrits van den Ende and de Hoog [43]. For the identification of the majority of strains internal transcribed spacers 1 and 2 including the 5.8S rDNA (ITS) were used, and for fungi belonging to the genus *Cladosporium* the gene encoding for actin (*act*) was used. These genes were PCR amplified and Sanger sequenced with the following primer sets: ITS1/ITS4 (ITS) [44], and ACT-512F/ACT-738R (*act*) [45]. The fungi were identified by comparison to the sequences of the most similar type strains and other closely related strains stored in the non-redundant GenBank nucleotide database with the blast algorithm [46]. All DNA sequences of the isolated strains from this study were deposited in the GenBank database: OQ376720‒ OQ376732 (ITS rDNA), OQ401817‒ OQ401829 (actin).

### 2.4 Visual appearance and microscopic observation

To capture high-quality images of the wood samples, an office scanner Bizhub C258 from Konica Minolta was used, with a resolution of 600 dpi. For microscopic observations, the Keyence VHX-6000 digital microscope (Keyence, Osaka, Japan) was utilised, which allowed for detailed imaging at magnifications of 50× and 500×. The microscope employed full ring illumination, and the intensity was optimised to ensure optimal image quality and eliminate saturated pixels. Additionally, high magnification images at 500× were captured using 3D depth reconstruction mode, providing a more detailed and comprehensive view of the samples.

### 2.5 Gloss and colour measurement

To evaluate the gloss and colour changes of the investigated wood specimens, a portable ERICHSEN Colour and Gloss Unit SPEKTROMASTER 565-45 (ERICHSEN GmbH & Co. KG, Germany) was employed. The gloss and colour were measured at five randomly selected spots along the longitudinal direction of each wood specimen. Colour changes were evaluated using the CIE Lab* colour space system, which uses three parameters to express colour: L* (lightness), a* (red-green tone), and b* (yellow-blue tone). The standard observer angle was set at 10°, and illuminant D65 was used. The total colour change ΔE was calculated according to Equation (2):

(2) $$\Delta E=\sqrt{{\Delta L}^{*2}+{\Delta a}^{*2}+{\Delta b}^{*2}}$$

where ΔL*, Δa*, Δb* correspond to differences between colour coordinate values measured at the given time and referenced to the corresponding value of initial colour.

### 2.6 Wettability

Dynamic contact angle with distilled water was measured on the optical tensiometer Attension Theta Flex Auto 4 (Biolin Scientific, Gothenburg, Sweden). Five measurements were performed on each specimen, using the sessile drop method. The nominal volume of each drop was 4 μL. The volume was precisely controlled by both droplet dispenser and a digital image analysis tool integrated with instrument software. The measurement of the drop contour was initiated at the moment of drop contact with the assessed sample surface. The droplet image acquisition lasted for 20 s. Images were post-processed with the tensiometer proprietary software (One Attension v.4.0.5). The contact angle was determined by implementing the Laplace equation. The contact angle observed at the third second of the test was assumed as a representative quantifier. Five replicate measurements were averaged to reduce the scatter of results. The range of observed values (minimum–maximum) was used to define the variability and reliability of the contact angle assessment.

## 3 Results

### 3.1 Weather data

Table 1 presents the weather data that were averaged in two-week intervals. During the testing period, the average temperature in Izola, Slovenia was higher than that in Skellefteå, Sweden. In Izola, the temperature ranged from 8.7 to 34.7 °C, with the highest average temperature of 25.0 °C recorded in July (weeks 3–4). On the other hand, the temperature in Skellefteå was in the range of 0.5–30.1 °C, with the highest average temperature of approximately 21.4 °C recorded in July (weeks 0–2). It is noteworthy that the relative humidity in Skellefteå ranged from 30% to 100%, while in Izola, it ranged from 22% to 100%. The highest average relative humidity in Izola was recorded in July (weeks 0–2) at 66.2%, whereas in Skellefteå, it was approximately 84.3% in August (weeks 7–8) and September (weeks 11–12). The maximum wind speed in Skellefteå was 43.2 km/h in July (weeks 3–4), while in Izola, it was 34.2 km/h in September (weeks 5–6). The total duration of wind blowing towards the south was higher in Izola than in Skelleftea. Specifically, in Izola the duration was more than 200 h every 2 weeks, while in Skelleftea it was significantly lower. The highest cumulative precipitation recorded in Izola at each two-week stage of the experiment duration was 67.2 mm and was noticed in September (weeks 11–12). Correspondingly, 132.9 mm of rain which is nearly double the amount was recorded in Skellefteå during weeks 7–8 (August).

**Table 1:** Weather data during the test averaged in two-week intervals. () presents the number of days with precipitation over 0.25 mm.

Period [weeks]	Temperature [°C]		RH [%]		Total wind blowing towards the south (hrs)		Wind speed [km/h]		Total precipitation [mm]	
	Izola	Skellefteå	Izola	Skellefteå	Izola	Skellefteå	Izola	Skellefteå	Izola	Skellefteå
0 to 2	24.1	21.4	66.2	62.4	239	150	9.5	12.1	52.7 (7)	15.9 (3)
3 to 4	25.0	17.0	65.2	65.7	229	62	9.5	15.2	0.6 (1)	20.8 (3)
5 to 6	24.9	15.7	65.0	73.1	226	115	10.6	10.4	35.3 (6)	14.3 (2)
7 to 8	23.0	12.1	58.8	84.3	239	81	10.7	12.0	39.3 (5)	132.9 (9)
9 to 10	19.8	10.7	60.1	72.1	211	61	9.8	11.8	3.8 (2)	9.9 (3)
11 to 12	19.3	7.5	73.4	84.3	276	222	9.2	10.5	67.2 (7)	72.3 (6)

The analysis of the Scheffer Climate Index (SCI) values showed that Skellefteå had an SCI value of 27.1, which indicates relatively unfavourable conditions for above-ground wood decay in this location. Conversely, the SCI value for Izola was 45.7, indicating moderately favourable conditions for wood decay. Even the difference in the SCI values is apparent; the number of rainy days was very similar at the exposure site in Slovenia and Sweden. It corresponded to 28 and 27 days, respectively. The relative humidity promoting the growth of moulds exceeded RH > 80% for 24% of the exposure time in Izola and 46% in Skellefteå.

### 3.2 Fungal analysis

#### 3.2.1 Culturable fungal composition

Through DNA sequence analyses, fungal strains belonging to the genera *Aureobasidium* and *Cladosporium* were detected on the weathered wood surfaces at both locations. Specifically, *Aureobasidium* and *Cladosporium*. These included *Aureobasidium melanogenum*, *A. pullulans, Cladosporium allicinum*, *C. pseudocladosporioides, C. crousii, and C. westerdijkiae* (Table 2). Additionally, other fungi were detected during the study, including *Sydowia polyspora, Endoconidioma populi, Lithohypha guttulata, Pseudotaeniolina globos*a, and *Stachybotrys* sp. However, no evidence of wood decay fungi was observed.

**Table 2:** List of analyzed strains subjected to DNA sequence analyses and morphological studies.

EXF-number	Identification	Geographical origin	Genebank accession number
EXF-16440	*Aureobasidium melanogenum*	Slovenia, Izola	ITS: OQ376720
EXF-16450	*Aureobasidium melanogenum*	Slovenia, Izola	ITS: OQ376723
EXF-16452	*Aureobasidium pullulans*	Slovenia, Izola	ITS: OQ376724
EXF-16462	*Aureobasidium melanogenum*	Sweden, Skelleftea	ITS: OQ376731
EXF-16438	*Cladosporium allicinum*	Slovenia, Izola	act: OQ401817
EXF-16439	*Cladosporium pseudocladosporioides*	Slovenia, Izola	act: OQ401826
EXF-16441	*Cladosporium crousii*	Slovenia, Izola	act: OQ401829
EXF-16442	*Cladosporium pseudocladosporioides*	Slovenia, Izola	act: OQ401825
EXF-16444	*Cladosporium allicinum*	Slovenia, Izola	act: OQ401819
EXF-16445	*Cladosporium pseudocladosporioides*	Slovenia, Izola	act: OQ401822
EXF-16447	*Cladosporium pseudocladosporioides*	Slovenia, Izola	act: OQ401821
EXF-16448	*Cladosporium allicinum*	Slovenia, Izola	act: OQ401818
EXF-16449	*Cladosporium pseudocladosporioides*	Slovenia, Izola	act: OQ401824
EXF-16451	*Cladosporium* sp.	Slovenia, Izola	act: OQ401827
EXF-16458	*Cladosporium allicinum*	Sweden, Skelleftea	act: OQ401820
EXF-16459	*Cladosporium pseudocladosporioides*	Sweden, Skelleftea	act: OQ401823
EXF-16460	*Cladosporium westerdijkiae*	Sweden, Skelleftea	act: OQ401828
EXF-16461	*Endoconidioma populi*	Sweden, Skelleftea	ITS: OQ376730
EXF-16457	*Exophiala xenobiotica*	Sweden, Skelleftea	ITS: OQ376729
EXF-16454	*Lithohypha guttulata*	Slovenia, Izola	ITS: OQ376726
EXF-16456	*Lithohypha guttulata*	Slovenia, Izola	ITS: OQ376728
EXF-16443	*Phoma herbarum*	Slovenia, Izola	ITS: OQ376721
EXF-16453	*Pseudotaeniolina globosa*	Slovenia, Izola	ITS: OQ376725
EXF-16455	*Pseudotaeniolina globosa*	Slovenia, Izola	ITS: OQ376727
EXF-16446	*Stachybotrys* sp.	Slovenia, Izola	ITS: OQ376722
EXF-16463	*Sydowia polyspora*	Sweden, Skelleftea	ITS: OQ376732

#### 3.2.2 Effects of weather conditions on fungal colonisation on wood surface

**Fig. 2:**
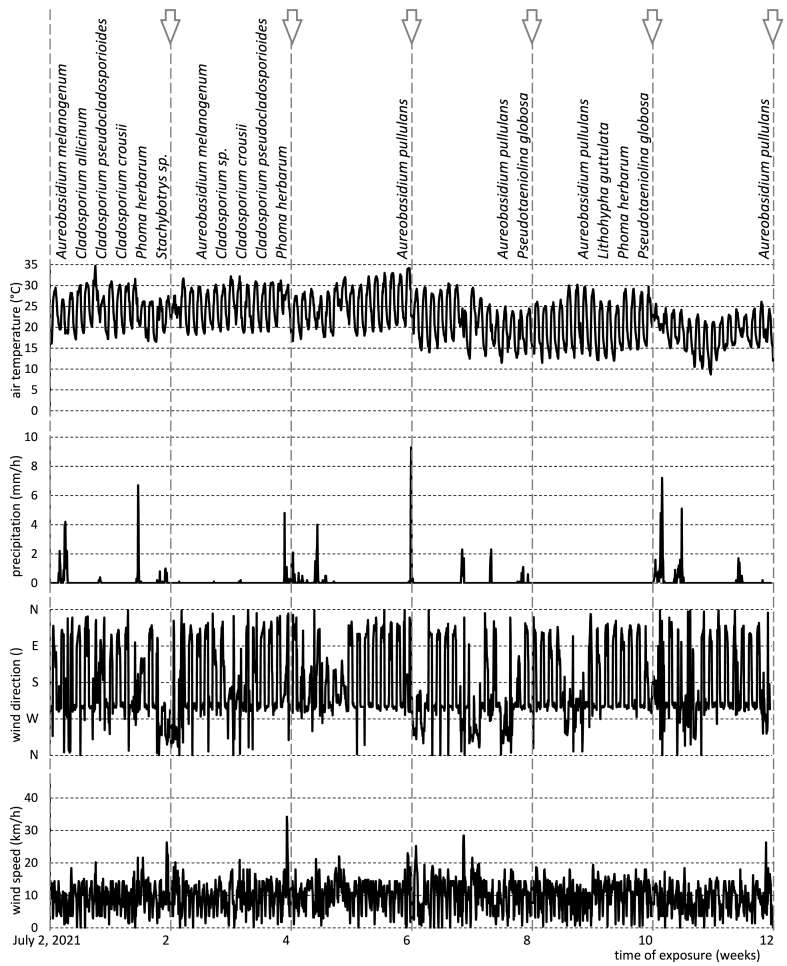
Fungal species colonised on Scots pine wood in correlation with weather condition during exposure in Izola, Slovenia. Weather conditions are presented hourly.

**Fig. 3:**
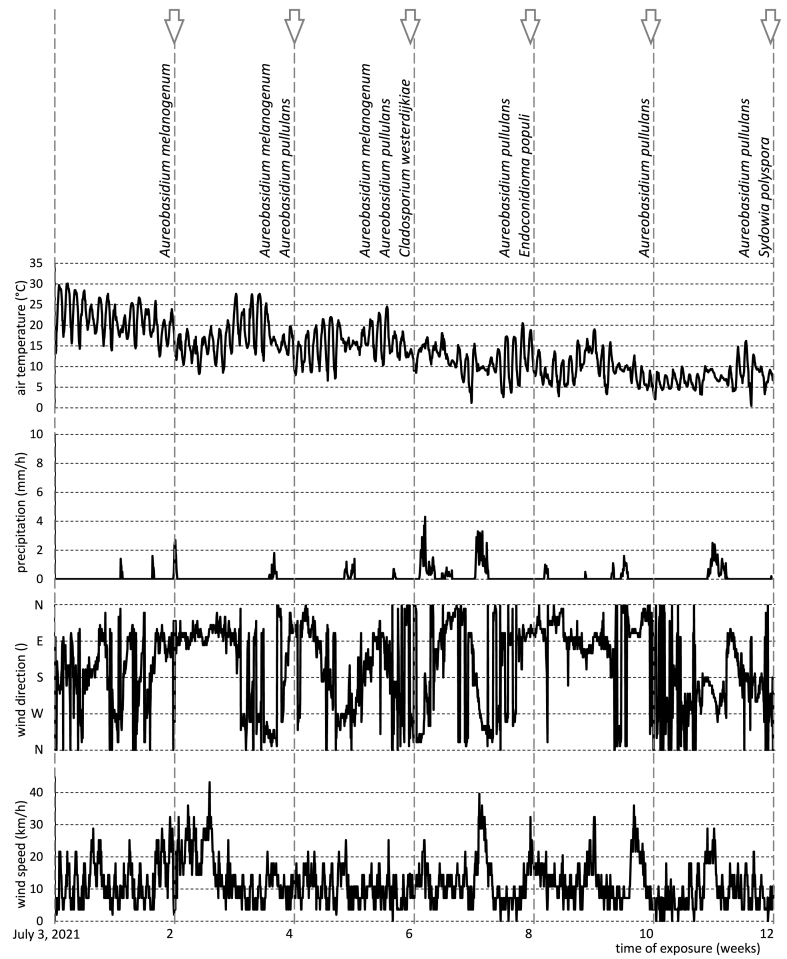
Fungal species colonised on Scots pine wood in correlation with weather condition during exposure in Skellefteå, Sweden. Weather conditions are presented hourly.

Fig. 2, Fig. 3 depict the fungal species identified on the surface of Scots pine wood in relation to hourly weather conditions recorded during exposure in Izola and Skellefteå, respectively. More detailed observations including timeline analysis and replicate samples are presented in Table 3. In Izola, the most abundant fungal species was recorded in July (week 0–2), when the average temperature was 24.1 °C, the average relative humidity was above 65%, the average wind speed was 9.5 km/h, and the total precipitation was 52.7 mm with 7 days of precipitation over 0.25 mm (Table 1). In Skellefteå, the highest variety of fungi was noticed at the beginning of August (week 5–6), when the average temperature was 15.7 °C, the average relative humidity was 73.1%, the average wind speed was 10.4 km/h, and the total precipitation was 14.3 mm with 2 days of precipitation over 0.25 mm (Table 1).

**Table 3:** *Timeline analysis and replicates of fungal strains detected in Izola* () *and Skellefteå, Sweden* (●).

	Time of exposure to natural weathering (weeks)					
**Fungal species**	2	4	6	8	10	12
*Aureobasidium melanogenum*	● ●	● ●	● ● ●			
*Aureobasidium pullulans*		● ● ●	● ●	● ●	● ● ●	● ● ●
*Cladosporium allicinum*						
*Cladosporium crousii*						
*Cladosporium pseudocladosporioides*						
*Cladosporium* sp.						
*Cladosporium westerdijkiae*			●			
*Endoconidioma carpetanum*				● ●		
*Lithohypha guttulata*						
*Phoma herbarum*						
*Pseudotaeniolina globosa*						
*Stachybotrys* sp.						
*Sydowia polyspora*						● ●

During the weathering campaign, the composition of culturable fungal species on wood samples showed high fluctuations (Table 3). In terms of fungal species diversity on weathered wood, a higher number of fungal species were found in Izola, which has a humid subtropical climate (Cfa) with hot, humid summers, compared to Skellefteå, which has a subarctic or boreal climate (Dfc) with short, cool summers. Fungi in the genera *Aureobasidium* and *Cladosporium* were most frequently found on weathered wood surfaces in both locations. *Aureobasidium pullulans* was particularly abundant during weeks 3–12 of exposure in Skellefteå.

### 3.3 Surface visual appeal

**Fig. 4:**
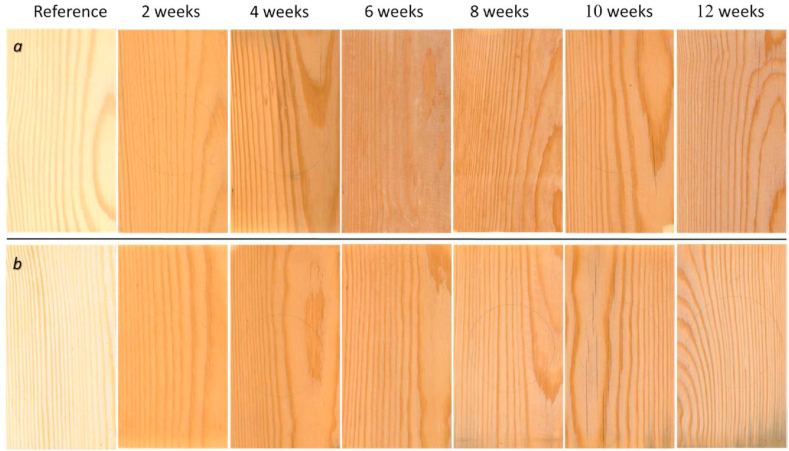
Appearance of samples during weathering test in Izola, Slovenia (a) and in Skellefteå, Sweden (b).

The surface of weathered wood at both locations become darker after the initial two weeks of exposure, and then slightly lighter after eight weeks until the end of the weathering process. In Skellefteå, a dark-grey colour due to the presence of fungi became visible to the naked eye after eight weeks of exposure. The majority of fungal colonisation consisted of melanised fungi such as *A. pullulans, E. populi*, and *S. polyspora*, which were mostly found on the bottom part of the wood samples. This may be due to the extensive accumulation of moisture within the wood samples, which can occur as a result of the “moisture trap” effect and capillary uptake of water in the bottom part of vertically exposed samples. Cracks began to form after two weeks of exposure to natural weathering at both study locations, and larger cracks, raised grains, and eroded surface marks appeared after 10 weeks. Images of the investigated wood samples are shown in Fig. 4.

### 3.4 Microscopic observations

**Fig. 5:**
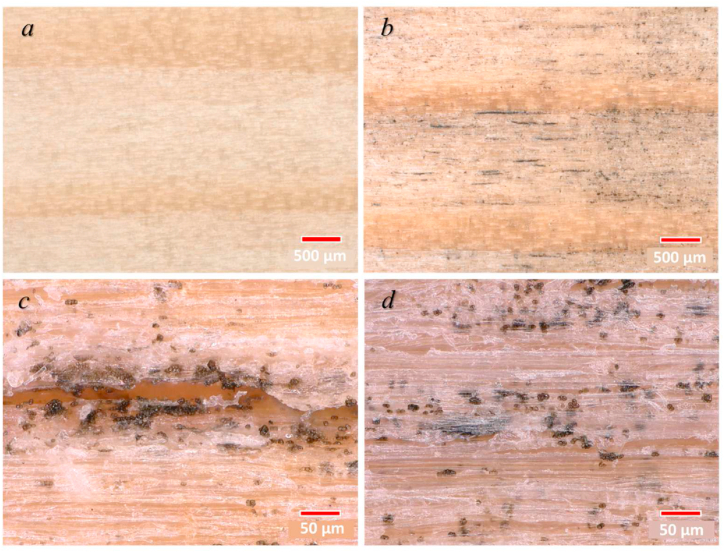
Microscopic images with 50× magnification: reference (not weathered) wood (a), wood surface colonised by fungi after 12 weeks of exposure in Skellefteå, Sweden (b). Details of accumulation of fungal colonies in the crack (c) and on rough surface of earlywood of weathered samples (d) observed with 500× magnification**.**

Microscopic images of weathered wood samples revealed the presence of microcracks as well as signs of fungal colonisation on the surface of weathered wood (Fig. 5). Visual representation of the contrasting characteristics of earlywood and latewood in their original state, prior to undergoing weathering processes is shown in Fig. 5a. The development of fungi until the 12th week was observed mostly on the earlywood zone (Fig. 5b, d). Highest accumulation of fungal colonies was noticed within (micro)cracks on the wood surface (Fig. 5c).

### 3.5 Surface colour

**Fig. 6:**
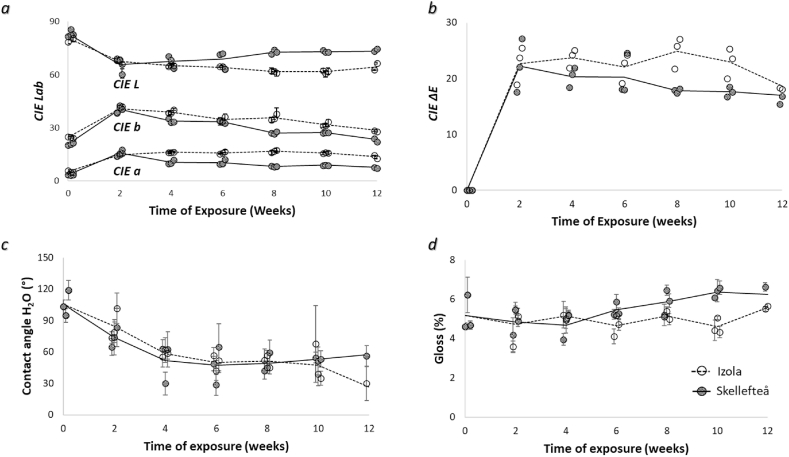
CIE L*a*b* colour coordinates, CIE L*, CIE a*, CIE b* (a), and CIE ΔE (b), change in contact angle (c) and changes in gloss assessed in the longitudinal direction (d) of Scots pine wood as a function of exposure time. Note: Error bars correspond to the range (minimum–maximum) of results.

The changes in CIE Lab* colour coordinates measured on the surface of Scots pine during the natural weathering test at both locations are presented in Fig. 6a and b. The average CIE L* of the Scots pine surfaces decreased during the first two weeks of exposure, and for the samples from Izola, it remained relatively constant until the end of the test, while for the samples exposed in Skellefteå, the average CIE L* slightly increased after the initial drop and then stayed relatively constant (Fig. 6a). The decreasing CIE L* of samples at both locations resulted in the apparent darkening of the weathered wood, as is clearly visible in Fig. 4. The average CIE a* colour coordinate for samples increased at both locations during the initial stage, up until two weeks of exposure. After that, it became relatively steady for the samples in Izola, while it gradually decreased for those in Skellefteå (Fig. 6a). Similarly, the average CIE b* coordinates of wood surfaces at both locations increased in the initial stage, for up to two weeks of exposure, and then steadily decreased after that (Fig. 6a). The total colour change (CIE ΔE) of Scots pine wood at both locations followed a similar trend as CIE a* and CIE b*, with an initial rapid rise followed by a slow drop in the CIE ΔE value (Fig. 6b). However, it can be noted that the overall colour change was steadily higher for the Izola samples compared to the samples in Skellefteå. The colour differences diminished after prolonged exposure of 12 weeks, where both long-term exposed samples appeared relatively similar (Fig. 4).

## 4 Observed

### 4.1 Wettability

The trend of changes in wettability induced by the natural weathering process for samples at both study locations is shown in Fig. 6c. A similar trend, showing a decrease in contact angle θ, was observed for samples from Izola and Skellefteå during the initial stage of weathering until the 8th week. However, after that period, the wettability slightly decreased for the wood samples exposed at Skellefteå, while a different trend was noticed for samples from Izola where θ continued to decrease until the end of the experiment.

### 4.2 Gloss evaluation

The trend of change in gloss measured on Scots pine wood samples was slightly different between Izola and Skellefteå, (Fig. 6d). The surface gloss remained relatively constant for Izola samples with minor fluctuations in values until the 10th week of exposure. A slight increase in the gloss value was noticed afterward, even if it is impossible to interpret it as a stable trend. In case of samples from Skellefteå the steady increase in gloss value was noticed after a minor decrease at the initial exposure period.

## 5 Discussions

Identified fungi detected in both locations represent blue stain and mould fungi from the phylum *Ascomycota* which are known as the primary coloniser on wood surfaces [47,48] and are normally found on painted surfaces [49,50] and building materials [51]. However, the fungi identified in this study might be limited to hydrophilic and mesophilic strains, as MEA was used as the culture media. Certain fungi such as thermophilic or xerophilic fungi thrive in extreme environments and may not grow well under these conditions [52]. However, MEA is the most frequently recommended culture medium used in aerobiological studies, given the wide range of fungal genera found in the air [53]. Further studies using different culture media and conditions may be necessary to identify a broader range of fungal species present in the studied samples.

The results of this study indicate that the climate conditions in Izola are more favourable for fungal colonisation of wood compared to Skellefteå, as evidenced by the higher SCI value observed in Izola. This indicates a greater risk of above-ground wood decay in Izola as compared to Skellefteå. These results are consistent with the decay risks in climatic exposure zones in Europe established by VTT, where Skellefteå, situated in the boreal climate zone, has a lower decay risk compared to Izola, which falls under the Mediterranean climate zone [26]. It is noteworthy that no evidence of wood decay fungi was detected in either location during the study. The SCI values, which provide a site-specific assessment of the relative hazard of above-ground wood decay, should be interpreted as an indicator of the potential for fungal colonisation rather than a definitive measure of actual decay. Additionally, the climatic exposure zones established by VTT provide relative values for decay risks in different parts of Europe, which is not directly related to mould growth. However, since similar environmental factors such as temperature and humidity can affect both mould growth and wood decay, it is possible that the conditions for mould growth in Izola may also be more favourable than in Skellefteå. These findings suggest that environmental factors may play a role in both processes, even though the intensity and conditions required for mould growth can differ significantly from those necessary for wood decay.

Based on the study results, temperature and humidity were identified as possible factors in the colonisation of fungi on Scots pine wood. The average temperature in Izola (ranging from 19.3 to 24.1 °C) falls within the favourable range for fungal growth, as defined by Viitanen, 1996 and Viitanen, 1997 [54,55]. Despite the relatively low humidity levels (ranging from 59% to 73%), fungi were still able to colonize the wood. Optimum temperature for blue stain growth on wood is between 18 and 29 °C. The moisture span typically ranges from fibre saturation to near maximum moisture content (*U*_max_). For many species, the optimum moisture content falls between 30 and 120% [11]. Moulds can grow between 0 and 50 °C, with an optimal temperature of 20–35 °C [54]. The critical limit for mould growth on wood is around 75–80% RH, but growth mostly requires a higher RH of around 90%. Fluctuating humidity can retard growth, and higher humidity (RH > 95% at 20–40 °C) can promote rapid growth [55]. These conditions also correlate positively with the concentration of fungal spores in the air [16,17,56].

The dominance of *Cladosporium* in July on the Izola samples can be attributed to its abundance in the atmosphere during this period [[57], [58], [59], [60]] in conjunction with the favourable weather conditions. It's important to note that the fungal species identified up to the 4th week, including *Cladosporium*, were not present in week 6 and afterward in August and September. Instead, other fungal species such as *A. pullulans, P. globose, L. guttulata, and P. herbarum* were detected. The disappearance of some fungal species in August and September could be attributed to detachment of fungal conidia by thunderstorms and wind-driven drops of rain [61] that occurred in Izola during August (Fig. 2). Rainfall can wash away fungal spores from the air resulting in even lower concentrations of culturable fungi in the atmosphere in the days following a rain [62,63]. Similarly, strong winds and rainfall during weeks 7–10 possibly facilitated the transport and germination of spores from new fungal species on the surface of wood samples exposed in Izola [[64], [65], [66], [67]]. Wind and rain also play an important role in the dispersal and concentration of fungal spores in the atmosphere [[68], [69], [70]]. Although high relative humidity is more favourable for fungal growth, low relative humidity in combination with high wind speed may enhance the liberation of fungal spores in the air [71]. Conversely, Lin and Li (2000) [72], Lin and Li, 2000 observed a strong negative correlation between fungal concentration and wind speed under 5 m/s. Nevertheless, the concentration of fungal spores increased when the wind speed was higher than 5 m/s. Atmospheric fungal spore levels exhibit a statistically significant positive correlation with the direction of the wind [73]. Wind direction may contribute to the dispersion and release of fungal spores onto the wood surface. Specifically, in Izola, where the total period of wind blowing towards the south was higher than in Skelletea, there was a greater probability of fungal spores being dispersed onto the wood surface.

Fungi from genus *Aureobasidium* were detected on the wood surfaces exposed to natural weathering in Skellefteå during the entire period of exposure. *A. pullulans* was most frequently observed, particularly during the moderately low temperature period from 3 to 12 weeks of exposure. The predominance of *A. pullulans* during this period can be attributed to its ability to survive under low temperatures [74]. The presence of *A. pullulans* in August and September (6–12 weeks) is also consistent with previous observations [75]. The highly successful colonisation of this species on wood surfaces at both study locations can be attributed to its ability to survive under a wide range of climatic conditions, low nutrient requirements, its ability to metabolise lignin breakdown products, its capacity to withstand desiccation and high temperatures [4,5,7,[76], [77], [78]], and its effective antagonistic action against a wide range of fungi [[79], [80], [81]], including wood blue staining fungi [82]. Moreover, the ability of *A. pullulans* to colonize the surface of painted wood [[83], [84], [85]], wood coated with semi-clear coating [86], ceramic roof [87], and oil treated wood [88,89] has been reported. Apart from the other characteristics mentioned above, the extracellular polysaccharides (EPS) synthetised by this microorganism can enhance adhesion of fungal spores to the surface of the substrate [90,91]. Consequently, *A. pullulans* was always present on the wood surface in Skellefteå, even during the period of strong wind and heavy rain (Fig. 3). It was particularly noticeable during week 7–8 when total precipitation was more than 130 mm with 9 days of precipitation above 0.25 mm. Similarly, total precipitation during week 11–12 was 67.2 mm with 7 days of precipitation above 0.25 mm (Table 1). It resulted in an abundance of *A. pullulans* on the assessed wood surfaces.

*Cladosporium* is also one of the most common fungal genera detected in the atmosphere in different parts of the world [17,56,60,63,69,72]. The predominance of *Cladosporium* observed on the surface of wood samples at the beginning of the natural weathering test in this study can be attributed to their abundance in the atmosphere and their ability to grow in all regions, on broad types of substrates, and weather conditions [17,62].

Colour changes indicate the physical-chemical changes on the wood surface induced by the action of diverse weather conditions during exposure. Rapidly decreasing CIE L* of Scots pine during the initial exposure phase indicates darkening of the wood surface due to the accumulation of degradation products of lignin caused by the UV light and solar irradiation [4,5,92,93] in combination with the presence of melanised fungi, such as *Aureobasidium*, *Cladosporium,* S*tachybotrys*, and *Phoma* on the wood surface [4,94]. Research has reported strong correlations between the mould rating and ΔL* on wood that has been exposed to natural weathering in Ås, Norway [95]. The initial increase in CIE b* is associated with the degradation of lignin [[96], [97], [98]] among other factors. The subsequent decrease in CIE b* is associated with the rainwater gradually leaching the decomposed lignin components and its extractives [98]. The change in CIE a* values is associated with chemical changes to the chromophore groups present in some wood extractive components [99]. The rate of photooxidation on wood surfaces is accelerated by higher air temperatures [4,100], which may explain the higher total colour changes observed in the Izola samples compared to those in Skellefteå. The colour changes of Scots pine sapwood exposed to natural weathering appear to be strongly influenced by weather conditions, as indicated by the consistent trend in colour changes observed within the same climate zone. A 3-month outdoor exposure study on Scots pine sapwood conducted by Poohphajai et al., 2021 [31] in San Michèle, Italy, which falls under the humid subtropical climate zone, Cfa [35] showed similar values of CIE Lab* colour coordinates to the results obtained from our own study conducted in Izola, which is also within the same climate zone. Furthermore, a decrease in lightness on the surfaces of Scots pine sapwood exposed to natural weathering over a 13-week period in Ås, Norway, which falls under the subarctic or boreal climate zone, Dfc [35] as observed by Lie et al., 2019 [95] was consistent with the results obtained from our study conducted in the same climate zone in Skellefteå, Sweden. Additionally, darkening of the wood surface caused by surface mould growth was visually detected after several weeks in both locations within this climate zone. The findings support the notion that weather conditions have an impact on the colour changes of Scots pine sapwood.

Earlywood cells are characterised by thinner cell walls and broader cell lumens when compared to latewood cells, making it easier for fungal hyphae to penetrate towards the wood bulk. The presence of grooves and pits within the rough surfaces of earlywood (Fig. 6d.) offers multiple attachment options for microorganisms and provides them with protection from the elevated air pressure and resulting shear forces in the surrounding environment [[101], [102], [103], [104], [105]]. Highest accumulation of fungal colonies was noticed within (micro)cracks on the wood surface (Fig. 6c.). In addition to providing mechanical protection, rough surfaces and cracks can also concentrate nutrients and moisture more easily, creating more favourable conditions for fungal attachment and growth [106].

The main reason for the increase in wettability of wood surfaces caused by weathering is the removal of extractive components as well as lignin derivatives during exposure to biotic conditions [4,107]. The increase in wettability of the weathered wood surface accelerates further degradation of the subsurface leading to an extended surface erosion and corrosion. Moreover, elevated surface moisture content as a result of high surface wetting promotes the loosening of cellulose fibres. The removal or washing out of the fibres by wind driven rain triggers the creation of micro-cracks, resulting in an increase in (micro)roughness [108]. On the contrary, the presence of *A. pullulans* as well as other fungi on the weathered wood surface may fill the micro-voids and other irregularities on the wood surface. It reduces the surface roughness and, eventually, decreases wettability [31]. Therefore, the slight decrease in the wettability of the samples in Skellefteå can be connected to the presence of *A. pullulans* and *S. polyspora* colonies. Both species accumulated in the cracks and rough surface of earlywood which were identified on the high magnification microscopic images (Fig. 5). Similar trend of changes in wettability of weathered wood surfaces were previously reported [107].

The change in gloss of weathered wood is generally attributed to surface mechanical abrasion and chemical erosion. Some researchers have reported an increase in gloss values on wood surfaces exposed to artificial weathering under controlled exposure conditions [109]. Other studies have reported a reduction in glossiness of wood surfaces [[110], [111], [112]]. The removal of early wood tracheids results in elevated microroughness, while the exposed middle lamellae surfaces reflect light more specularly than the cut/broken cell wall. An increase in the gloss value might also be associated with the removal, first of extractives, and then of wood degradation products [4]. It is difficult to isolate the direct effect of fungi on weathered surfaces, as the overall change in gloss is the result of all the above-mentioned factors and mechanisms.

## 6 Conclusions

The results obtained confirm the overall hypothesis linking intensity of wood weathering and wood surface colonisation by fungi. The kinetics of the deterioration of natural wood surfaces by abiotic factors as well as the activity of fungi, both depend on the specific weather conditions in different (European) climate zones. Certain species such as *A pullulans* seem to be prevalent as primary colonisers independent of the geographical location. Understanding the interaction of fungi with the substrate is necessary in order to identity fungal strains that might be used as a protective layer on building materials. Such an approach allows for the development of novel bioinspired protection coatings based on optimised fungal biofilm working in synergy and not against nature.

## Author contribution statement

Faksawat Poohphajai: Conceived and designed the experiments; Performed the experiments; Analyzed and interpreted the data; Contributed reagents, materials, analysis tools or data; Wrote the paper, Revised the paper.

Olena Myronycheva: Conceived and designed the experiments; Performed the experiments; Contributed reagents, materials, analysis tools or data; Wrote the paper; Revised the paper.

Olov Karlsson: Revised the paper.

Lauri Rautkari: Revised the paper.

Jakub Sandak: Conceived and designed the experiments; Performed the experiments; Analyzed and interpreted the data; Contributed reagents, materials, analysis tools or data; Wrote the paper; Revised the paper.

Ana Gubenšek: Analyzed and interpreted the data; Revised the paper.

Polona Zalar: Analyzed and interpreted the data; Contributed reagents, materials, analysis tools or data; Revised the paper.

Nina Gunde-Cimerman: Contributed reagents, materials, analysis tools or data; Revised the paper.

Anna Sandak: Conceived and designed the experiments; Performed the experiments; Analyzed and interpreted the data; Contributed reagents, materials, analysis tools or data; Wrote the paper, Revised the paper.

## Data availability statement

The dataset used for analysis in this study is available at Zenodo.org Open Access depository [113].

## Declaration of competing interest

The authors declare that they have no known competing financial interests or personal relationships that could have appeared to influence the work reported in this paper.
